# A 52-Year-Old Female with a Hoarse Voice and Tingling in the Hand

**DOI:** 10.1371/journal.pmed.0040029

**Published:** 2007-03-06

**Authors:** Salman Razvi, Petros Perros

## DESCRIPTION of CASE

**Figure 1:**
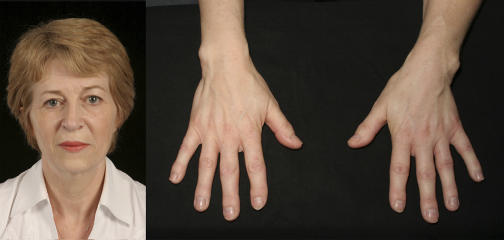
Appearance of the Patient The patient's facial appearance shows some broadening of the nose and supra orbital ridges (left). At right, the patient's large hands are shown.

A 52-year-old female presented to an otolaryngologist with a hoarse voice. An endocrine opinion was sought to exclude an underlying endocrinopathy. She had been investigated three years previously by a neurologist for right-sided facial pain. A magnetic resonance imaging (MRI) scan had shown a small “cyst” in the pituitary gland. She had been reassured that this was a coincidental finding. The patient did not volunteer any specific symptoms and had no complaints other than a hoarse voice. Her appearance is shown in Figure 1.

### How Should a Patient with Suspected Pituitary Disease Be Worked Up?

The above scenario is increasingly common as a result of the advent of powerful imaging techniques such as MRI and computerised tomography scanning, which identify “coincidental” pituitary abnormalities in a significant minority of the adult population. Nonetheless, when such lesions are identified they warrant further characterisation.

The clinician confronted with such a case should ask the following questions:

- Is there evidence of over secretion of pituitary hormone(s)?
- Is there evidence of deficiency of pituitary hormones?
- Is there evidence of pressure on structures surrounding the pituitary fossa?

The commonest cause of excess pituitary hormone secretion is a prolactinoma. It usually presents with galactorrhoea, oligo- or amenorrhoea, and hirsutism in premenopausal women and with hypogonadism in men. Cushing disease and acromegaly are less common and gonadotrophin-secreting adenomas and thyrotrophinomas are rare. Mild hyperprolactinaemia is commonly found in association with large pituitary tumours and is usually due to distortion of the pituitary stalk. This “disconnection” syndrome must be distinguished from a true prolactin-producing adenoma, as the treatment of the latter (with dopamine agonist drugs) is radically different from that of other types of pituitary adenomas.

Hypopituitarism can present with features of hypogonadism (amenorrhoea, loss of body hair, loss of libido and impotence), hypothyroidism, and hypoadrenalism. The latter, in addition to symptoms of hypocortisolism (tiredness, dizziness, fatigue) may also be associated with loss of ability to tan and generalised hypopigmentation.

Expansion of a pituitary mass superiorly can compress the optic chiasm, thus giving rise to bitemporal hemianopia. Lateral extension can compress cranial nerve III, IV, or VI. Occasionally pituitary tumours can bleed or infarct to cause pituitary apoplexy (sudden headache, photophobia, cranial nerve palsies, and collapse due to hypoadrenalism and in some cases hypoglycaemia). Investigations include pituitary function tests, formal visual field assessment, and in some cases further more detailed imaging.

Despite the absence of spontaneous symptoms, the patient admitted to increasing hand and shoe size when specifically asked, associated with a hoarse voice of three years duration. She had also suffered from frontal headaches as well as tingling of the right index finger. Previously, she had surgery for temporomandibular joint dysfunction.

On closer inspection, there was a suggestion of some broadening of the nose and prominence of the lips, rather large hands, and increased skin thickness (Figure 1). Visual fields were normal and Tinel's sign was positive. Blood pressure (BP) was 160/96 mm Hg. Blood tests showed normal urea, creatinine and electrolytes, fasting glucose of 6.6 mmol/l (normal range, <5.6 mmol/l), prolactin 330 imu/l (normal <550 imu/l), and thyroid stimulating hormone (TSH) of 1.4 mIU/l (normal 0.3–4.7 mIU/l) with free T4 of 16 pmol/l (normal 11–23 pmol/l).

### What Is the Reason for the Tingling of the Right Index Finger?

The sensory disturbance in the distribution of the median nerve is typical of right carpal tunnel syndrome (CTS). CTS typically presents as pain in the medial three fingers radiating into the arm, worsening during the night.

Thickening of tendons or synovitis in the carpal tunnel area causes CTS. The history, in this patient, is typical of CTS and is characterised by pain, tingling, and numbness in the median nerve distribution. This patient does not have hypothyroidism (normal thyroid hormone and serum TSH levels) or diabetes mellitus (although she has impaired fasting glycaemia), was not pregnant or obese, and did not have a history or clinical manifestations of rheumatoid arthritis. The cause could be idiopathic, but the other features in the history and examination point towards endocrinopathy as being the underlying aetiology. The diagnosis of CTS can be confirmed by an electromyogram.

### Is the Combination of High Blood Pressure and High Fasting Glucose Levels Common?

Hypertension associated with problems with glucose metabolism is a common feature usually seen in people with the metabolic syndrome. Less commonly, it can be seen in endocrine diseases like Cushing syndrome and acromegaly. The reason for hypertension and hyperglycaemia in Cushing syndrome is due to the excess glucocorticoid levels whereas in acromegaly, it is due partly to the direct action of excess growth hormone (GH) on sodium retention and increased insulin resistance, respectively [1].

### What Is the Next Step?

The symptoms of increasing soft tissue size, CTS, hypertension, hyperglycaemia, and temporomandibular joint dysfunction make acromegaly a possible diagnosis (Box 1). The duration of symptoms as well as the previous surgery for temporomandibular joint dysfunction suggests that the disease has been active for at least three years [2]. The usual (in 98% of cases) cause of acromegaly is a GH-secreting pituitary adenoma, usually a macro-adenoma (>1 cm). Other rare causes are pituitary carcinoma or GH-releasing hormone tumours.

**Box 1. Symptoms and Signs of Acromegaly**

**Symptoms due to direct effect of the tumour**

- Headache
- Visual impairment
- Hypopituitarism
- Hyperprolactinaemia

**Symptoms due to GH excess**

- Soft tissue enlargement
- Headache
- Increased skin tags
- Increased sweating
- Acanthosis nigricans

**Cardiovascular features**

- Hypertension
- Biventricular hypertrophy
- Diastolic dysfunction at rest and systolic dysfunction on effort
- Endothelial dysfunction

**Metabolic and other endocrine features**

- Impaired fasting glycaemia
- Impaired glucose tolerance
- Type 2 diabetes mellitus
- Insulin resistance
- Dyslipidaemia
- Multinodular goitre
- Hypercalciuria
- Hyperparathyroidism

**Musculoskeletal features**

- Joint stiffness
- Arthropathy and osteoarthritis
- Carpal tunnel syndrome
- Osteopenia

**Respiratory disease**

- Upper airway obstruction and snoring
- Macroglossia
- Obstructive sleep apnoea
- Thickened vocal chords and hoarse voice

**Neoplastic disease**

- Colorectal polyps and cancer
- Probable breast, prostate, and thyroid cancer

Baseline GH levels are of limited value in diagnosing acromegaly since GH is normally secreted episodically, and high levels can also be seen in pregnancy, puberty, in response to pain, stress, malnutrition, and after a prolonged fast. The diagnosis can be confirmed by measuring GH levels in response to an oral glucose tolerance test (OGTT) (Box 2 outlines the protocol for OGTT to diagnose acromegaly). Insulin-like growth factor 1 (IGF 1) is a protein modulated by GH and produced in many body tissues, primarily in the liver. Serum levels of IGF 1 are fairly stable throughout the day and correlate closely with mean GH serum concentration.

**Box 2. Protocol for Oral Glucose Tolerance and GH Suppression Test for Diagnosis of Acromegaly**

- Fast the patient from midnight.
- Insert venous cannula prior to basal sample.
- Administer oral 75 grams glucose at time 0 (just prior to basal blood sample) and collect blood samples for GH and glucose at 30-minute intervals up to 120 minutes.
- Measure IGF 1 level with basal sample.

Further investigation and management of patients with suspected pituitary disease should be undertaken in specialised endocrine centres, to which such patients should be referred.

The patient's random GH level was 55 mu/l (0–20) along with an elevated IGF 1 level of 78 nmol/l (age-matched normal range being 12–44). She then proceeded on to an OGTT with glucose and GH level measurements; the results are outlined in Table 1.

**Table 1:**
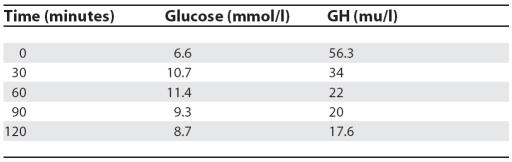
Oral Glucose Tolerance Test with Growth Hormone Results

**Figure 2:**
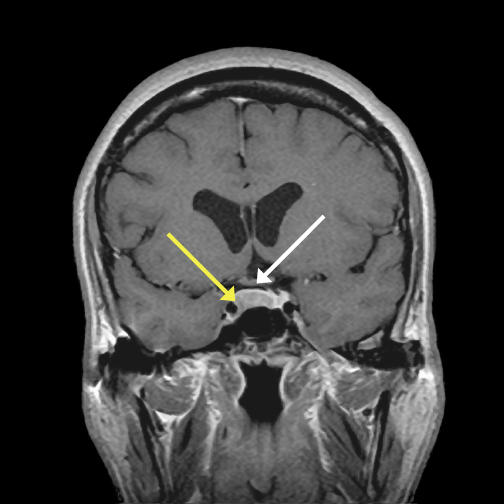
MRI Studies of the Patient's Pituitary Gland MRI of the pituitary gland showing a non-enhancing lesion occupying the bulk of the pituitary fossa (yellow arrow). The remaining normal pituitary is pushed to the left, as is the pituitary stalk. The optic chiasm can be seen just superior to the pituitary gland (white arrow).

The OGTT confirms failure of GH to suppress to levels below 2 mu/l (<1 mcg/l) as well as impaired fasting glycaemia and impaired glucose tolerance. Other tests of anterior pituitary function (prolactin, thyroid function tests, luteinising hormone, and follicle-stimulating hormone) as well as dynamic testing of adrenal reserve in response to synthetic ACTH (short synacthen test) were normal. MRI studies of the pituitary gland revealed a pituitary micro-adenoma (Figure 2).

### Should Imaging Precede the Biochemical Investigations?

This patient's case highlights the dilemma clinicians are facing today as a result of the increasing use, availability, and precision of imaging techniques. She had a MRI scan previously that detected a pituitary tumour. Pituitary tumours that are detected coincidentally (so called “incidentalomas”) are not uncommon with reports of prevalence of up to 10% in adults. Given the limitations of diagnostic testing, biochemical screening requires sufficiently high pre-test probability to make it effective. Apart from the cost factor, an imaging-first principle in any field in medicine that relies on biochemical proof of disease leads to a cascade of tests and pursuit of false-positive results. Therefore, appropriate clinical evaluation should precede biochemical investigations, which then may need to be confirmed by imaging techniques [3].

### Should This Patient Be Treated?

The pathologic effects of increased GH levels are progressive and are associated with increased morbidity and mortality. People with acromegaly have a 2- to 4-fold increase in mortality rate, mostly due to cardiovascular disease [4]. Many of the soft tissue overgrowth features as well as the increased cardiovascular risk can be reversed, at least partly for soft tissue and completely for cardiovascular disease, by appropriate therapy [5]. Appropriate therapy may also reduce or even prevent mass effects caused by tumour growth in the pituitary area. It is therefore important that diagnosis and treatment are instituted quite promptly.

### What Treatment Modalities Are Available?

The underlying treatment strategy of acromegaly is to remove the pituitary tumour (especially macro-adenomas causing mass effects), normalise GH and IGF 1 levels whilst preserving normal residual pituitary function, and relieve symptoms. Medical, surgical, and radiotherapeutic means or, more commonly, some combination of the three can be used to try and achieve this. Currently five different modalities of treatment are available, although surgery remains the treatment of choice. Table 2 outlines the advantages and limitations of each modality.

**Table 2:**
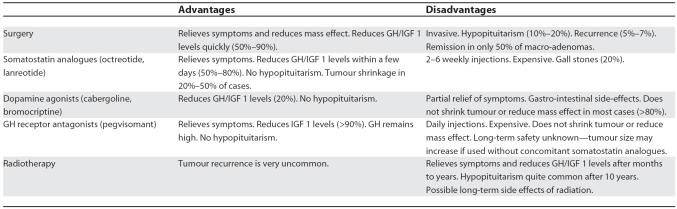
Advantages and Limitations of Different Modalities of Treatment for Acromegaly

In this patient, it was decided that it would be prudent to try and regress some of the soft tissue changes of acromegaly to minimise the risks of intubation during anaesthesia (although this approach is empirical and not widely practised). This was achieved by a somatostatin receptor analogue therapy, given every month. This reduced GH levels to 35 mu/l and IGF 1 levels to 56 nmol/l.

After four monthly injections of long-acting somatostatin receptor analogue therapy, the patient went on to have an endoscopic pituitary adenomectomy. Histology of the resected adenoma revealed a GH cell pituitary adenoma.

### How Should Response to Surgery Be Assessed?

The currently accepted criteria for cure of acromegaly is a random or mean GH level of <5 mu/l or a nadir GH level of less than 2 mu/l during an OGTT and a serum IGF 1 level within the age-adjusted normal range [6]. The patient had an OGTT eight weeks after surgery as well as tests of anterior pituitary function. Nadir GH levels were 8.6 mu/l with a slightly raised IGF 1 level of 51 nmol/l, signifying partial resolution of acromegaly. Other anterior pituitary function tests were normal. Repeat MRI scan showed no definite target for further surgical intervention.

She was then started on somatostatin analogue therapy to achieve better GH level control. This reduced mean GH levels to 2.9 mu/l and normalised IGF 1 levels to 32 nmol/l. After discussion with the patient, it was decided not to proceed with radiotherapy since the acromegaly was well controlled with medical therapy and due to the high probability of hypopituitarism associated with radiotherapy. She remains asymptomatic and is under regular endocrine follow-up.

## DISCUSSION

Acromegaly is a rare condition with prevalence rates of 60 per million population. The condition can cause significant morbidity and mortality but may present with vague features; therefore a high index of clinical suspicion is warranted. The average delay in diagnosis after onset of the disease is about eight years. It can cause hypertension and disorders of glucose metabolism, which are increasing in prevalence, as well as soft tissue enlargement, which may take years to be noticed. The increasing use of imaging techniques has invented a new disorder—the incidentaloma. When incidentalomas are found in the pituitary, a thorough history and examination is required with relevant biochemical tests. The biochemical diagnostic criteria for acromegaly have changed over the past decade, bringing the GH diagnostic threshold to lower levels. The advent of better and more sensitive GH assays and better imaging have helped to diagnose more subtle forms of the disease, which would not have been picked up earlier, and may therefore hopefully reduce morbidity and mortality. Furthermore, newer treatment options, whilst expanding the therapeutic armour, also pose a challenge to the treating endocrinologist in deciding what best suits the patient.

**Key Learning Points**

- Acromegaly is a rare but easily diagnosed condition that can cause significant morbidity and mortality.
- Increasing use of imaging has led to an increase of incidental findings—the “incidentaloma”.
- Symptoms of acromegaly may persist for many years before the diagnosis is made.
- Newer modalities of GH assays and treatment have led to earlier diagnosis and may reduce symptoms and mortality.

## Abbreviations

CTS: carpal tunnel syndrome
GH: growth hormone
IGF 1: insulin-like growth factor 1
MRI: magnetic resonance imaging
OGTT: oral glucose tolerance test
TSH: thyroid stimulating hormone
